# Treatment-related benefit and satisfaction in patients with Fabry disease in France: insight into patients’ expectations and preferences from the prospective, non-interventional SATIS-Fab study

**DOI:** 10.1186/s13023-026-04285-7

**Published:** 2026-04-01

**Authors:** Olivier Lidove, Agathe Masseau, Grégory Pugnet, Didier Lacombe, Bertrand Dussol, Soumeya Bekri, Albert Hagège, Caroline Martinez, Yann Fardini, Alain Fouilhoux, Esther Noël

**Affiliations:** 1 Department of Internal Medicine, GH Diaconesses Croix Saint-Simon Centre de Référence Maladies Lysosomales (CRML), Filière G2M, 125 rue d’Avron, Paris, 75020 France; 2 Normandie Univ, UNIROUEN, AIMS, SysMedLab, Rouen, France; 3 CHU de Nantes, Site Hôtel Dieu, Nantes, France; 4 Department of Internal Medicine and Clinical Immunology, CHU de Toulouse Rangueil, Toulouse, France; 5 CHU de Bordeaux, INSERM U1211, Bordeaux University, Bordeaux, France; 6 APHM Hôpital de la Conception, Marseille, France; 7 Normandie Univ, UNIROUEN, AIMS, SysMedLab, Department of Metabolic Biochemistry, Referral Center for Lysosomal Diseases, Filière G2M, CHU Rouen, Rouen, France; 8 Hôpital Européen Georges-Pompidou AP-HP, Paris, France; 9 Amicus Therapeutics, Paris, France; 10 Soladis Clinical Studies, Roubaix, France; 11 Centre de Référence Maladies Héréditaires du Métabolisme Filière G2M, Hospices Civils de Lyon, Hôpital Femme Mère Enfant, Bron, France; 12 CHU de Strasbourg, Strasbourg, France

**Keywords:** Fabry disease, Migalastat, Enzyme replacement therapy, Treatment expectations, Treatment satisfaction, Patient Benefit Index, Non-interventional study, France, Quality of life

## Abstract

**Background:**

Fabry disease (FD) is a progressive X-linked lysosomal disorder caused by *GLA* variants resulting in deficient α-galactosidase A enzyme activity, glycolipid accumulation, and multisystemic dysfunction. Approved treatments include intravenous enzyme replacement therapy (ERT) or the oral small-molecule chaperone migalastat.

**Methods:**

SATIS-Fab was a 2-year, prospective, longitudinal, multicentre, non-interventional, non-comparative study conducted in France. Enrolled patients were aged ≥ 16 years, had FD (migalastat-amenable variant), and were receiving/planned to initiate ERT or migalastat. The primary endpoint during follow-up (6-monthly assessments) was the FD-specific Patient Benefit Index (PBI), derived from the French-validated Patient Needs Questionnaire-Fabry and corresponding Patient Benefit Questionnaire. Secondary endpoints included the Treatment Satisfaction Questionnaire for Medication-9 (TSQM-9).

**Results:**

Of 69 enrolled patients (mean age 51.7 years; 63.8% male; 60.9% late-onset phenotype), 39/69 were already receiving migalastat and 17/69 ERT. At the baseline visit, 82.6% (57/69) were prescribed migalastat and 17.4% (12/69) ERT. Twelve-month PBI remained stable (mean [SD] 2.36 [0.91] and 2.49 [0.94] at months 12 [*n* = 55] and 24 [*n* = 47], respectively; *P* = 0.173). Mean (SD) 24-month PBI was 2.43 (0.92), and 92.3% of patients (48/52) had a clinically relevant treatment benefit (24-month PBI ≥ 1). Correlations between 24-month PBI and TSQM-9 effectiveness, convenience, and global satisfaction domain scores were 0.63, 0.43, and 0.61, respectively (all *P* < 0.0001; *post hoc* analysis); these results confirm the external validity of the PBI. Ten patients switched from ERT to migalastat at/after the baseline visit and before month 24; none switched from migalastat to ERT. For those with available data (*n* = 8), mean (SD) 12-month PBI increased by 1.14 (0.96) after switching (*P* = 0.008) and TSQM-9 domain scores improved (all *P* < 0.05).

**Conclusions:**

Patients with FD, mostly receiving migalastat, reported a relatively high level of treatment benefit that remained stable over 2 years. Switching from ERT to migalastat was associated with significantly increased treatment benefit. The FD-specific PBI could support shared decision making about treatment.

**Trial registration:**

NCT04043273 (registered 31 July 2019).

**Supplementary Information:**

The online version contains supplementary material available at 10.1186/s13023-026-04285-7.

## Introduction

Fabry disease is a rare, progressive X-linked lysosomal disorder caused by variants of the *GLA* gene coding for the α-galactosidase A (α-gal A) enzyme [1–3]. This results in a functional deficiency in α-gal A activity and the progressive accumulation of glycolipids (predominantly globotriaosylceramide [Gb3] and globotriaosylsphingosine [lyso-Gb3]) in plasma and a wide range of cells and tissues throughout the body, which affects numerous organs and body systems leading to multisystemic dysfunction and a variety of clinical symptoms [1–3]. Signs and symptoms of Fabry disease can include peripheral nervous system dysregulation (such as neuropathic pain, heat/cold intolerance, and impaired sweating), progressive renal insufficiency, cardiac involvement (particularly cardiomyopathy and arrhythmias), cerebrovascular complications (including transient ischaemic attacks and ischaemic strokes), gastrointestinal issues, dermatological (angiokeratomas), and auditory and ocular symptoms [1–4]. The clinical presentation, severity, and age of onset of Fabry disease manifestations vary among individuals [1, 2]. Two distinct phenotypes are apparent: ‘classic’ (early-onset Fabry disease, which presents in childhood) and ‘later onset’ (more heterogeneous presentation in which some of the typical cardiac signs and symptoms, and sometimes reduced estimated glomerular filtration rate [eGFR], may not present until the fourth decade of life or later). Given the X-linked nature of the condition, manifestations of the disease are more variable in females than in males, although major organ involvement is still common in both [1–3, 5, 6]. The manifestations of Fabry disease can be life-threatening and have a negative impact on patients’ life expectancy and quality of life [1, 2, 7–10].

Current treatment options for Fabry disease in France include intravenous enzyme replacement therapy (ERT) with agalsidase alfa [11] or beta [12], or migalastat, an orally administered small-molecule chaperone therapy approved for the treatment of patients with a migalastat-amenable *GLA* variant [13, 14]. Fabry disease guidelines recommend that management should consider patient-reported outcomes [15, 16] and that treatment decisions should involve the patient and consider their preferences [16]. In addition, the French National Authority for Health (Haute Autorité de Santé) shared decision making report recommends a patient-centric approach to disease management, with the aim of improving patient safety, quality of care, and compliance [17, 18]. Generic patient-reported outcome (PRO) tools, however, do not provide disease-specific insight into the daily lives of patients and the impact of Fabry disease treatments.

The Patient Needs Questionnaire-Fabry (PNQ-Fabry) was developed by French patient associations and a board of French physicians with expertise in Fabry disease [18]. The development and validation followed international standards and best practice for PRO tools in rare diseases from the US Food and Drug Administration and European Medicines Agency [18]. Using the PNQ-Fabry, patients rank how important particular treatment needs and expectations are to them [18]. While this can help with shared decision making about treatment, it does not show how well those needs and expectations are being met by the prescribed treatment. To understand this, a Patient Benefit Questionnaire (PBQ) may be used; the questionnaire allows patients to report the extent to which the prescribed treatment helps with each of the needs and expectations in the corresponding, disease-specific PNQ. The PBQ responses can then be weighted according to the importance a patient has previously placed on each need and expectation; this calculation is referred to as the Patient Benefit Index (PBI) [19]. The PNQ–PBQ–PBI methodology was established in 2009 [20] and numerous disease-specific versions exist, including for many chronic skin diseases, asthma, and multiple sclerosis [21]. Repeated use of both the PNQ and PBQ during treatment makes it possible to assess whether a treatment continues to meet patients’ needs and expectations, even if those needs and expectations change over time. Consequentially, the assessments have the potential to enrich patient–physician conversations, particularly those around treatment adherence.

SATIS-Fab was a prospective, longitudinal, multicentre, non-interventional, non-comparative study (NCT04043273) in which treatment benefit in patients with Fabry disease in France was evaluated with reference to their treatment needs and expectations using the PNQ–PBQ–PBI methodology. It was hypothesized that needs and expectations are not similar for all patients and that distinct patient profiles exist. Clustering patients according to their PNQ-Fabry responses was the primary objective of a planned interim analysis of baseline data. The final analysis was conducted using data obtained during the 2-year follow-up phase, and the primary objective was to evaluate treatment benefit with reference to treatment needs and expectations using the PBI. Other PROs included satisfaction with, and preference for, Fabry disease treatment.

## Methods

### Study design

**Fig. 1:**
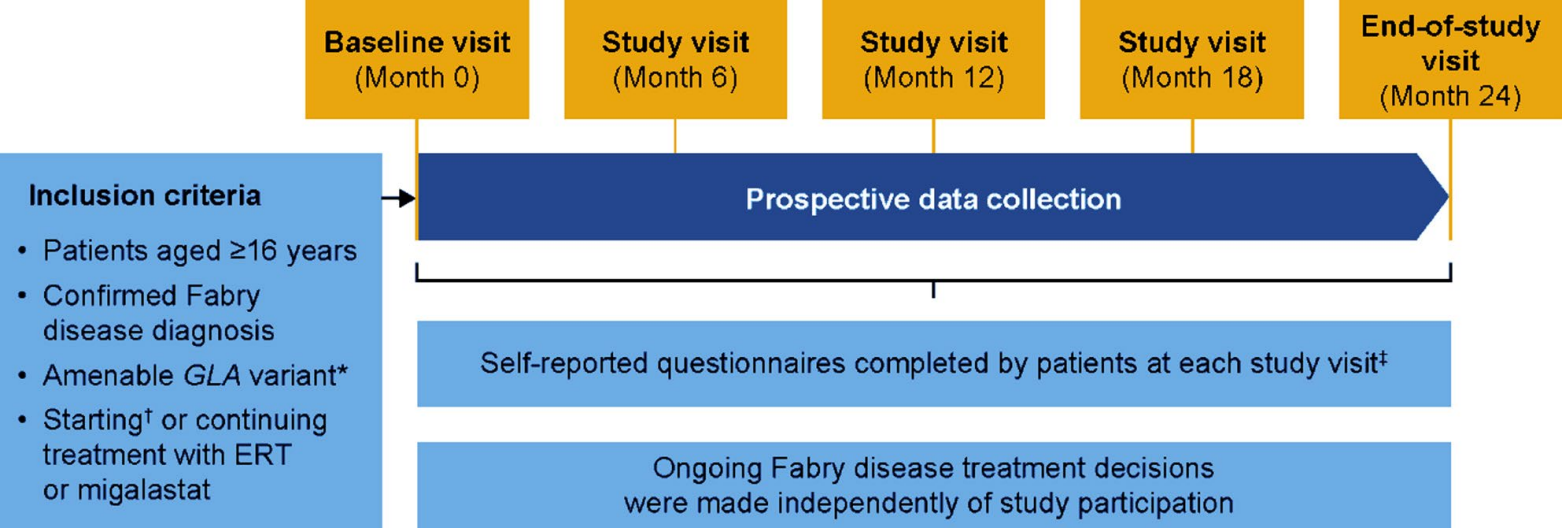
SATIS-Fab study design. As this was an observational study, the post-enrolment visit schedule could not be imposed and was left to physician choice. Physicians were instructed to send the self-report questionnaires to patients if no visit was planned or a planned visit did not take place. *All patients were required to have a migalastat-amenable *GLA* variant regardless of treatment received. ^†^Due to start ERT or migalastat within 2 months after study enrolment. ^‡^PNQ-Fabry, PBQ, TSQM-9, SF-36v2^®^, BPI and patient preference regarding ERT/migalastat; at the baseline visit, the PBQ, TSQM-9, and SF-36v2^®^ were completed only by patients currently receiving Fabry disease treatment (ERT or migalastat). MSSI was completed only at the baseline visit. BPI, Brief Pain Inventory; ERT, enzyme replacement therapy; MSSI, Mainz Severity Score Index; PBQ, Patient Benefit Questionnaire; PNQ-Fabry, Patient Needs Questionnaire-Fabry; SF-36v2^®^, Short Form-36 Health Survey version 2^®^; TSQM-9, Treatment Satisfaction Questionnaire for Medication-9

The SATIS-Fab study design is summarized in Fig. 1.

Patients were followed-up for 2 years, with a baseline (enrolment) visit, 6-monthly follow-up visits, and an end-of-study visit at 24 months. Treatment decisions were made independently of patients’ participation in the study; patients and physicians had free choice of starting or continuing ERT or migalastat, including switching treatment at any time during the study. A planned interim analysis of baseline data occurred when all patients had entered the study; the final analysis was conducted after the last patient’s end-of-study visit.

The study was conducted in accordance with the principles of the Declaration of Helsinki and the International Conference for Harmonisation Good Clinical Practice guidelines and in compliance with French laws and regulations. Ethics committee approval was obtained from the Comité de Protection des Personnes (CPP), Sud Méditerranée IV, Montpellier, France (initial approval date 9 October 2018, CPP reference 18 09 03). A non-opposition form was signed by adult participants or their legally acceptable representative; for minors, non-opposition from at least one parent or holder of parental authority was required.

### Patient population

Patients were eligible if they were aged ≥ 16 years, had a diagnosis of Fabry disease, and their physician had decided that they should start or continue ERT or migalastat treatment. If they were not currently treated, ERT or migalastat treatment was due to start within 2 months after study enrolment. All patients were also required to have a migalastat-amenable *GLA* variant. This was because patients’ preferences, needs and expectations regarding their treatment were integral to the study design. Having an amenable *GLA* variant meant that their treatment options were not restricted, as they could choose to be treated with either ERT or migalastat [13]. Patients were excluded if they were currently participating in an interventional clinical study (Category 1 or Category 2 interventional research according to the Jardé law classification in 1° and 2° of Article L. 1121-1 of the French Public Health Code).

### Study endpoints

Two sets of endpoints were planned, those relating to an interim analysis of baseline data and those relating to a final analysis of data obtained during follow-up.

For the interim analysis, the primary objective was to cluster patients based on their treatment-related needs and expectations identified through the PNQ-Fabry. In brief, responses to PNQ-Fabry questions were analysed using a principal component analysis (PCA). The primary endpoint was the components from the PCA (i.e. the components of the patients’ needs and expectations combined and ranked according to the amount of variance they explained). Patients were then clustered based on these components as described in the *Data analysis* section. Secondary endpoints included patient characteristics; patients’ preference regarding ERT or migalastat; treatment prescribed at the end of the baseline visit (ERT or migalastat); and, in currently treated patients, PBI (derived from the PNQ-Fabry and associated PBQ) and treatment satisfaction (as assessed by the TSQM-9 [22]).

For the final analysis, the primary endpoint was the PBI derived from the PNQ-Fabry and the PBQ. The PBI provides a weighted assessment of treatment benefits (from the PBQ) according to the importance placed on them by the patient (from the PNQ-Fabry). Secondary endpoints included: treatment satisfaction (TSQM-9); patients’ preference regarding ERT or migalastat; treatment prescribed at the end of each visit (ERT or migalastat); and evolution of treatment benefit (PBI) and satisfaction (TSQM-9) among patients switching treatment. Exploratory endpoints included the external validity of the PBQ and PBI, quality of life (assessed using Short Form-36 Health Survey version 2^®^ [23]), and clinical evolution (Fabry disease symptoms/complications, protocol-defined clinical events of interest, and pain).

### Assessments

The PBI was derived in a three-step process from two PROs. First, each patient reported the importance to them of each of the treatment needs and expectations in the PNQ-Fabry [18]. Second, patients reported, using the PBQ, how much their treatment helped with each need and expectation. Third, the PBI methodology outlined in the *Data analysis* section was used to weight the PBQ responses according to the relative importance each patient had assigned to treatment needs and expectations. Patients were invited to complete the PNQ-Fabry and PBQ every 6 months, and PBI scores were calculated for 6-, 12- and 24-month periods (see Supplementary Fig. 1). The PNQ-Fabry is a validated instrument comprising 26 treatment needs and expectations covering long-term efficacy, impact on daily activities, effectively treated symptoms, impact of treatment administration, and perception of mode of administration [18] (see Supplementary Table 1). The importance of each need and expectation is determined using a Likert scale (0 [not at all important] to 4 [very important], plus a ‘does not apply to me’ option). How much a treatment helps with each need and expectation (PBQ) is also determined using a Likert scale (0 [treatment did not help at all] to 4 [treatment helped very much]). In the PBI derived from the PNQ-Fabry and PBQ data, scores for each patient ranged from 0 (no patient-relevant benefit) to 4 (maximum patient-relevant benefit). PBI scores ≥ 1 were considered to represent a clinically relevant benefit and scores ≥ 3 a high benefit (thresholds previously applied in other disease-specific PBIs [19, 24–29]).

Additional assessments of patients’ attitudes to treatment comprised treatment satisfaction and treatment preference. Satisfaction was assessed using the TSQM-9, a validated self-administered questionnaire comprising nine questions grouped into three domains: effectiveness, convenience, and global satisfaction. Domain scores range from 0 to 100, with higher scores indicating higher satisfaction [22], and are derived by summing then transforming item scores [30]. Treatment preference was assessed using a single question (possible responses: ERT, migalastat, no preference).

Other assessments were as follows. Fabry disease symptoms at baseline were assessed using the Mainz Severity Score Index (MSSI; 0–76, with higher scores indicating more-severe symptoms) [31]. Pain severity and interference were assessed using the Brief Pain Inventory (BPI) scale (0–10, with 0 = least severe/no interference and 10 = most severe/interferes completely). Fabry disease symptoms/complications were recorded at baseline. New/changed Fabry disease symptoms/complications and new protocol-defined clinical events of interest (see Supplementary Table 2) were recorded during the study. Quality of life was assessed using the SF-36v2^®^ (see Supplementary Methods).

The results of laboratory and echocardiogram assessments were recorded if performed but were not study endpoints. Laboratory data were assigned only to a visit window, and no assessment dates were recorded (eGFR values were entered directly by investigators rather than being calculated centrally from serum creatinine levels). Echocardiogram data (specifically end-diastolic interventricular wall thickness and end-diastolic posterior wall thickness) were assigned to a visit window if the assessment took place within the previous 6 months.

### Data analysis

Data were recorded in electronic case report forms.

Sample size was determined based on the cluster analysis and the hypothesized number of components (six) resulting from the associated PCA (the primary endpoint in the interim analysis; i.e. 2^6^ = 64 patients).

The clustering of patients using baseline PNQ-Fabry responses was conducted as follows. First, responses to the 26 PNQ-Fabry questions were combined and ranked into components using a PCA. Second, a cluster analysis using Ward’s method [32] grouped patients based on these components. However, as this approach did not provide sufficient differentiation among patients (see Results), a *post hoc* K-means cluster analysis [33] was performed using the following baseline clinical characteristics: demographics (age, gender, body mass index, sick leave/disabled status related to Fabry disease), history of Fabry disease (time since diagnosis, phenotype), MSSI (general, neurologic, cardiovascular, and renal scores), BPI (pain severity, pain interference), SF-36v2^®^ (physical component score, mental component score), and Fabry disease signs and symptoms/complications. Treatment needs and expectations were described for each cluster using PNQ-Fabry responses.

The PBI for each patient was calculated as follows. First, each treatment need and expectation was weighted by dividing its Likert score by the sum of Likert scores for all treatment needs and expectations (responses of ‘does not apply to me’ were excluded from the calculations). Second, the weighted score for each treatment need and expectation was multiplied by its corresponding PBQ score and all results summed together to give a single PBI score. Mean PBI scores for the population were calculated using data from all patients who completed the questionnaires at each time point. The proportions of patients with PBI scores ≥ 1 (prespecified) and 3–4 (*post hoc*) had two-sided 95% confidence intervals (CIs) generated (Clopper–Pearson method). PBI scores were described overall and by *post hoc* cluster. No formal statistical comparison between treatments was planned since treatment allocation was not randomized. Trends over time in PBI scores were analysed using mixed models for repeated measures (Toeplitz covariance structure) with time (discrete variable) as a fixed effect, no random effects, and type III tests of fixed effects. For analyses of PBI scores by cluster group, the cluster group and an interaction term (time × cluster group) were added as fixed effects. *Post hoc* comparisons of 24-month PBI scores between clusters used a one-way analysis of variance (with no adjustments for baseline values) with the Tukey-Kramer *post hoc* test (residuals were checked for normality, and homogeneity of variance was checked using Bartlett’s test and Levene’s test).

Most secondary endpoints were analysed descriptively. The treatment prescribed (ERT or migalastat) and patients’ preference regarding ERT or migalastat were described at each study visit. The TSQM-9 effectiveness, convenience, and global satisfaction scores were summarized at each study visit, overall and by cluster group. For patients who switched treatment at least once during the study, PBI and TSQM-9 before and after switching were compared formally using a Wilcoxon signed-rank test (baseline and month 12 for switches before month 12; months 12 and 24 for switches at/after the month 12 visit). These time points were specified in the statistical analysis plan for the final analysis (superseding the protocol-specified before/6 months after time points) to account for data incompleteness at months 6 and 18 and provide a larger (more appropriate) time window.

Exploratory endpoints were summarized descriptively except for tests of the external validity of the PBQ (protocol-specified exploratory endpoint), 6-month PBI (specified in the statistical analysis plan for the final analysis), and 24-month PBI (*post hoc* analysis). These tests comprised Pearson correlations with the three TSQM-9 domain scores (treatment effectiveness, convenience, and global satisfaction).

## Results

### Study population

**Fig. 2:**
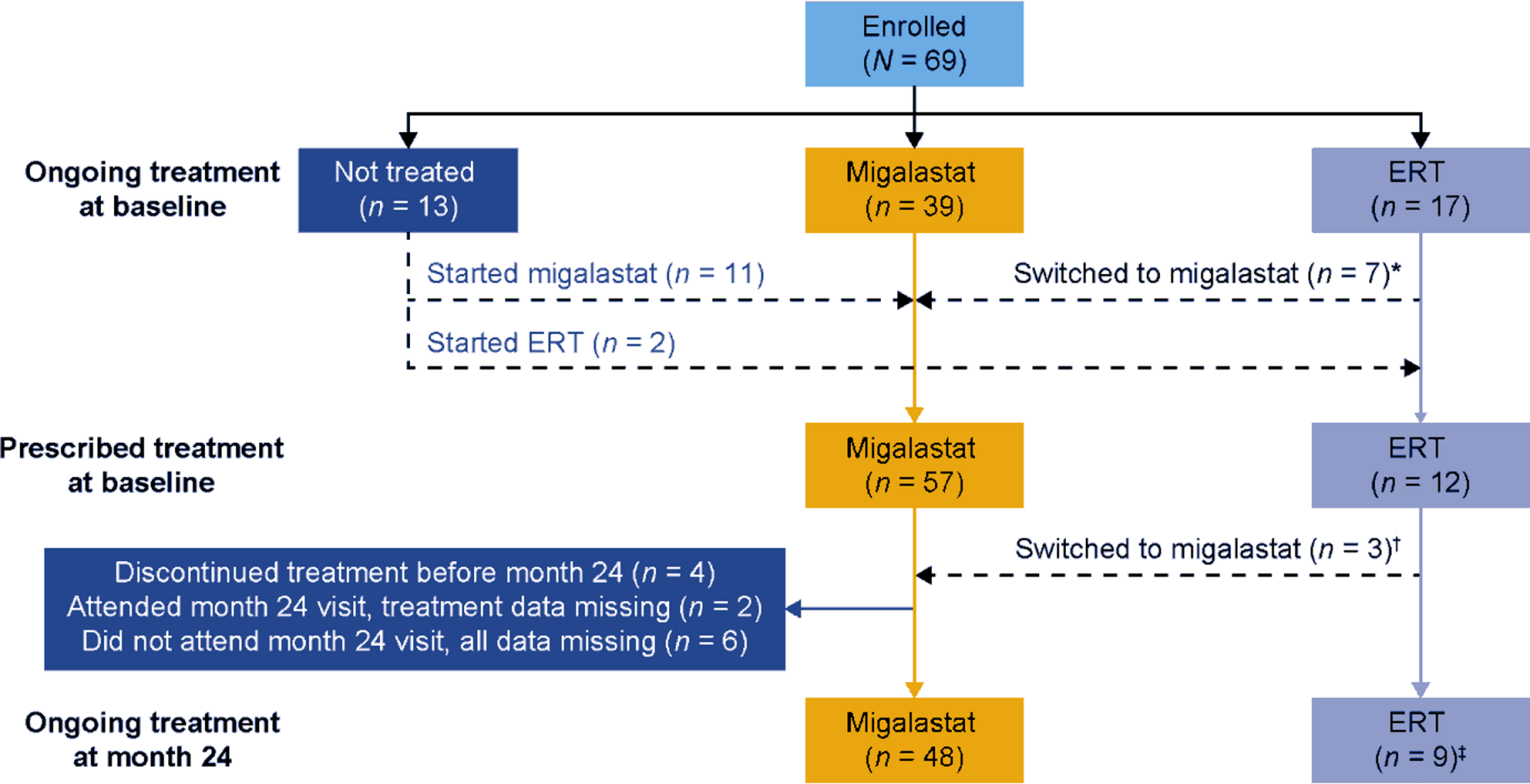
Fabry disease treatment during SATIS-Fab. *Owing to lack of efficacy (*n* = 1), at the patient’s request (*n* = 5), or for other reasons (difficult venous access, *n* = 1). ^†^At the patient’s request (*n* = 2) or for other reasons (patient’s request and poor tolerability, *n* = 1). ^‡^One patient was switched from ERT to migalastat *at* the month 24 visit at their request (shown here as receiving ongoing ERT treatment because migalastat treatment was planned to start *after* the month 24 visit). ERT, enzyme replacement therapy

**Fig. 3:**
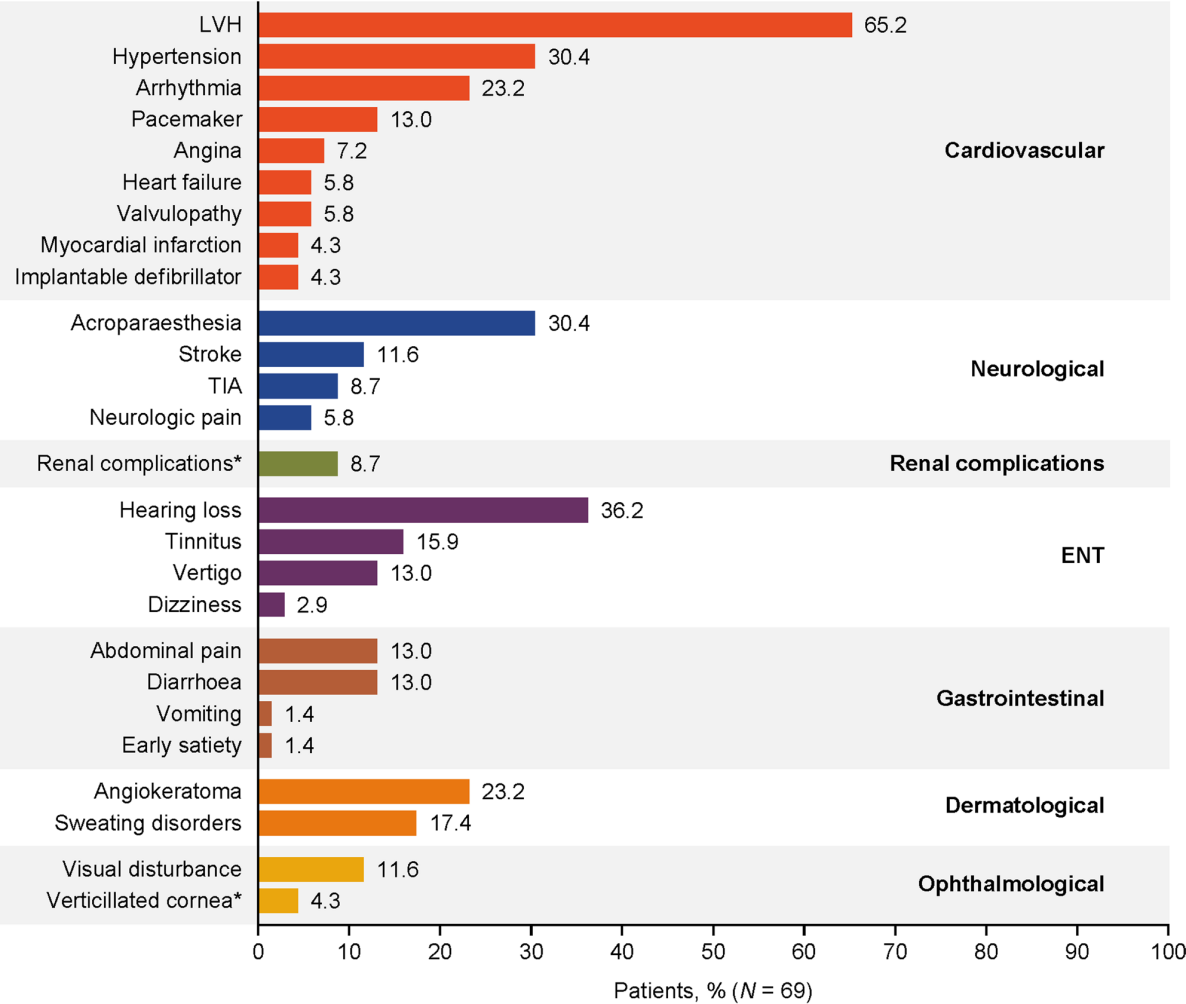
Fabry disease signs and symptoms/complications at baseline. Investigators recorded Fabry disease signs and symptoms/complications by selecting from protocol-defined terms or entering free text into a protocol-specified ‘other’ field, based on medical records and available laboratory data. Not shown are pulmonary (exertional dyspnoea in 11.6%, exercise intolerance in 10.1%, wheezing in 5.8%, and cough in 4.3% of patients), psychosocial (anxiety in 24.6%, depression in 15.9% and cognitive disorders in 4.3% of patients), and skeletal (osteopenia in 5.8% of patients) signs and symptoms/complications. *Coded by grouping all similar terms in the ‘other’ field. ENT, ear, nose, and throat; LVH, left ventricular hypertrophy; TIA, transient ischaemic attack

In total, 69 patients with Fabry disease (63.8% male) were enrolled at 16 centres in the SATIS-Fab study, which took place between 4 April 2019 (first patient, first visit) and 30 January 2023 (last patient, last visit). The population had a mean age of 51.7 years (range 21.0–76.0; Table 1). Clinically, the population was heterogeneous, comprising both classic and late-onset Fabry disease, and 81.2% (56/69) of patients were already receiving treatment at baseline (Fig. 2), with a mean MSSI score of 15.5 (range 0.0–32.0; Table 1). The most common α-gal A variants were p.Phe113Leu (17.4%) and p.Asn215Ser (17.4%). At baseline, 66 patients (95.7%) had at least one sign or symptom associated with Fabry disease; the most common individual signs or symptoms were left ventricular hypertrophy (LVH; reported by 45 patients, 65.2%), hearing loss (25 patients, 36.2%), acroparaesthesia (21 patients, 30.4%), and hypertension (21 patients, 30.4%; Fig. 3).

The study retention rate was good, with 50 (72.5%), 55 (79.7%), 49 (71.0%) and 59 (85.5%) of the 69 patients attending visits at months 6, 12, 18, and 24, respectively. The PRO questionnaire completion rate was also good – at least one questionnaire was completed by 98.6% of the cohort (68/69 patients) at baseline and by 75.4% (52/69) at month 24, with the lowest being 73.9% (51/69) at month 18.

**Table 1:** Baseline demographics and Fabry disease characteristics in the overall SATIS-Fab population

Parameter	Patients *N* = 69
**Mean age**,** years (SD); range**	51.7 (13.3); 21.0–76.0
**Male**, ***n* (%)**	44 (63.8)
**Mean BMI**, **kg/m**^**2**^** (SD)**	26.8 (5.7)
**Mean time since Fabry disease diagnosis**,** years (SD)**	6.2 (6.6)
**Fabry disease phenotype**,* *** n* (%)**	
Classic	23 (33.3)
Late-onset	42 (60.9)
Other	4 (5.8)
**Most common α-galactosidase A variants (> 15% of patients)**, ***n* (%)**	
p.Phe113Leu	12 (17.4)
p.Asn215Ser	12 (17.4)
**Comorbidities related to cardiovascular risk**,^†^ ***n* (%)**	
Hypertension	29 (42.0)
Dyslipidaemia	20 (29.0)
Diabetes	6 (8.7)
**MSSI scores **,^**‡**^** mean (SD) /median (range)**	
General	2.6 (2.6) / 2.0 (0–12.0)
Neurologic	3.5 (4.2) / 2.0 (0–16.0)
Cardiovascular	7.6 (5.9) / 9.0 (0–19.0)
Renal	1.8 (3.2) / 0.0 (0–18.0)
Total	15.5 (7.8) / 15.0 (0–32.0)
**BPI scores **,^**§**^** mean (SD) /median (range)**	
Pain severity (*n* = 65)	2.6 (2.0) / 2.5 (0–8.3)
Pain interference (*n* = 62)	2.7 (2.4) / 1.8 (0–8.4)
**Laboratory values**,^‖^** mean (SD)**	
Haemoglobin level, g/dL (*n* = 53)	14.3 (1.5)
Serum creatinine level, µmol/L (*n* = 58)	86.5 (34.0)
Proteinuria, mg/24 h (*n* = 38)	338.2 (421.1)
eGFR,^¶^ mL/min/1.73 m^2^ (*n* = 57)	85.7 (26.6)
**Proteinuria category**, ***n* (%)**	
< 300 mg/24 h	26 (68.4)
300–1,000 mg/24 h	10 (26.3)
> 1,000 mg/24 h	2 (5.3)
**eGFR**^¶^** category**, ***n* (%)**	
15–29 mL/min/1.73 m^2^	1 (1.8)**
30–59 mL/min/1.73 m^2^	9 (15.8)
60–89 mL/min/1.73 m^2^	24 (42.1)
≥ 90 mL/min/1.73 m^2^	23 (40.4)

*Patients were determined to have a classic or late-onset phenotype based on their *GLA* variant status (using a Fabry disease phenotype/genotype database in which variants were categorized as noted in this table) and at the discretion of the study principal investigator. ^†^Prespecified in the study protocol. ^‡^MSSI: composed of a total score (0–76 scale), with higher scores indicating more-severe symptoms; based on four subscores: general (0–18 scale), neurologic (0–20 scale), cardiovascular (0–20 scale), and renal (0–18 scale) [31]. ^§^BPI; consists of a pain severity score (0–10 scale) and a pain interference score (0–10 scale), with 10 being most severe or highest level of interference [8, 34]. ^‖^Data had been assigned to the baseline visit window; the assessment could have occurred at any time before the baseline visit as no dates were recorded. ^¶^eGFR values were entered directly into electronic case report forms by investigators rather than being calculated centrally from serum creatinine levels. **This patient was receiving ERT at baseline. BMI, body mass index; BPI, Brief Pain Inventory; eGFR_CKD−EPI_, estimated glomerular filtration rate – Chronic Kidney Disease Epidemiology Collaboration; ERT, enzyme replacement therapy; MSSI, Mainz Severity Score Index; SD, standard deviation

### Fabry disease treatment

At enrolment, 56.5% of patients (39/69) were already receiving migalastat treatment (mean [standard deviation (SD), range] time since migalastat start, 18.5 [16.2, 0–65.1] months). Smaller proportions were already receiving ERT (24.6%, 17/69; mean [SD, range] time since ERT start, 70.9 [65.2, 10.3–227.7] months) or were not receiving any treatment (18.8%, 13/69) for Fabry disease. At the baseline visit, 82.6% of patients (57/69) were prescribed migalastat and 17.4% (12/69) ERT; all untreated patients initiated treatment (Fig. 2) within 2 months after enrolment. During the study, ten patients switched from ERT to migalastat: seven at the baseline visit and three during follow-up. In addition, one patient planned to switch at the final study visit (month 24). No patients switched from migalastat to ERT. At the final study visit, 57 patients were recorded as receiving ongoing treatment (48/57 [84.2%] with migalastat and 9/57 [15.8%] with ERT, including the patient who planned to switch to migalastat), two attended but have missing data regarding ongoing treatment, four patients had already discontinued migalastat (one at month 6 [patient’s request] and three at month 12 [due to leukoencephalopathy (*n* = 1), pancreatitis (*n* = 1), and for an unknown reason (*n* = 1)]), and six did not attend (Fig. 2).

At baseline, most patients had a preference for migalastat treatment (72.5%; *n* = 50/69) rather than preferring ERT (13.0%; *n* = 9/69) or having no preference (14.5%; *n* = 10/69). At 24 months, this remained stable: most patients who completed the questionnaire still preferred migalastat treatment (73.7%; *n* = 42/57) rather than preferring ERT (8.8%; *n* = 5/57) or having no preference (17.5%; *n* = 10/57).

### PCA and cluster analyses

The PCA that preceded the planned cluster analysis identified seven components that explained 70% of PNQ-Fabry variance (labelled quality of social life, management of current symptoms, prevention of complications, resistance to physical exercise, easiness of treatment administration, fitness, and disruption of daily life by the treatment; Supplementary Table 3). Although four well-separated clusters were identified using these seven components, this approach did not provide sufficient differentiation because most patients were in the same large cluster (containing 83.6% [51/61] of those in the four clusters). As segmentation in this manner would only have minor implications and relevance for daily practice, an additional *post hoc* cluster analysis was performed using baseline clinical characteristics for all patients (*N* = 69; see Methods). Three well-differentiated clusters (clinical clusters) were identified with different profiles of signs/symptoms, symptom severities, and treatment needs and expectations (as described by the seven PNQ-Fabry components identified above). These clusters are described in the Supplementary Results including Supplementary Table 4. In brief (and by descending mean MSSI score), in Cluster 1 (*n* = 15, 53.3% classic phenotype, mean [SD] age 47.1 [8.4] years, mean [SD and range] MSSI total score 19.6 [9.3 and 1.0–32.0]), neurological symptoms dominated and two-thirds had LVH; patients placed greatest value on management of current symptoms, resistance to physical exercise, fitness, and quality of social life. Cluster 3 (*n* = 27, 25.9% classic, 64.3 [6.1] years, MSSI 17.4 [6.1 and 6.0–32.0]) primarily had cardiac signs/symptoms and placed greatest value on resistance to physical exercise, fitness, and (minimizing) disruption of daily life by treatment. Cluster 2 (*n* = 27, 29.6% classic, 41.7 [10.4] years, MSSI 11.3 [6.5 and 0.0–24.0]) placed greatest value on (minimizing) disruption of daily life by treatment.

### Treatment-related benefit and treatment satisfaction

The mean (SD) 24-month PBI score, which measures patient-reported benefits relative to needs and expectations expressed 24 months previously (here, at baseline), was 2.43 (0.92; *n* = 52). A clinically relevant treatment benefit (PBI score ≥ 1) was apparent for 92.3% (48/52; 95% CI 81.5–97.9%) of patients; high benefit (score of 3–4) was apparent for 30.8%.

**Fig. 4:**
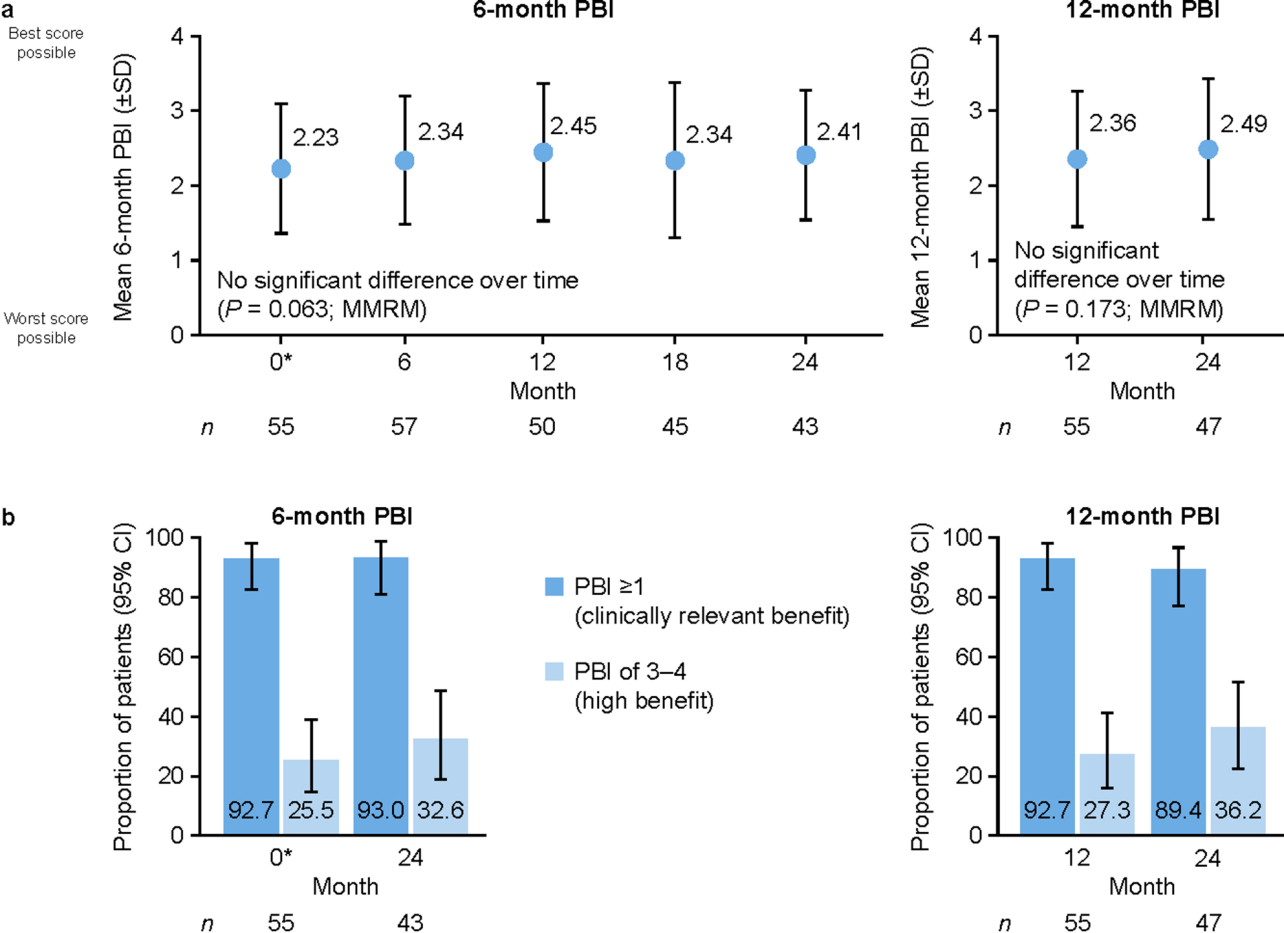
6-month and 12-month PBI over the study period. Panel **a** shows mean PBI scores during the study, with MMRMs indicating no significant trends over time. Panel **b** shows proportions of patients with clinically relevant or high benefit, with two-sided 95% CIs (Clopper–Pearson method; for intervening 6-monthly time points for the 6-month PBI, see Supplementary Fig. 2). *PBI for patients already treated before the baseline visit (calculated using PNQ-Fabry and PBQ responses at baseline; all other PBI values are calculated from PBQ responses at the time point shown and PNQ-Fabry responses from 6 months previously [6-month PBI] or 12 months previously [12-month PBI]; see Supplementary Fig. 1). CI, confidence interval; MMRM, mixed model for repeated measures; PBI, Patient Benefit Index; SD, standard deviation

Assessments of treatment benefit that accounted for changes in treatment needs and expectations during the study (6-month and 12-month PBI scores) were relatively stable, with no statistically significant differences over the study period (Fig. 4a). High proportions of patients were considered to have clinically relevant treatment benefit (Fig. 4b and Supplementary Fig. 2).

Overall, treatment satisfaction as measured by the TSQM-9 remained stable over the study period (Supplementary Fig. 3; numbers of patients with data were between 49 and 56). Mean effectiveness scores ranged from 65.7 (month 18) to 70.4 (month 6). Corresponding score ranges were 68.4 (baseline and month 18) to 72.7 (month 24) for global satisfaction and 67.1 (baseline) to 76.7 (month 12) for convenience.

In *post hoc* analyses, there were no statistically significant differences between clinical clusters in 24-month PBI (*P* = 0.155), 12-month PBI over time (*P* = 0.916), or 6-month PBI over time (*P* = 0.761). TSQM-9 scores were relatively stable over time, although mean scores for all three domains were numerically lower for Cluster 1 than for the other clusters.

### Treatment-related benefit and satisfaction after switching treatment

**Fig. 5:**
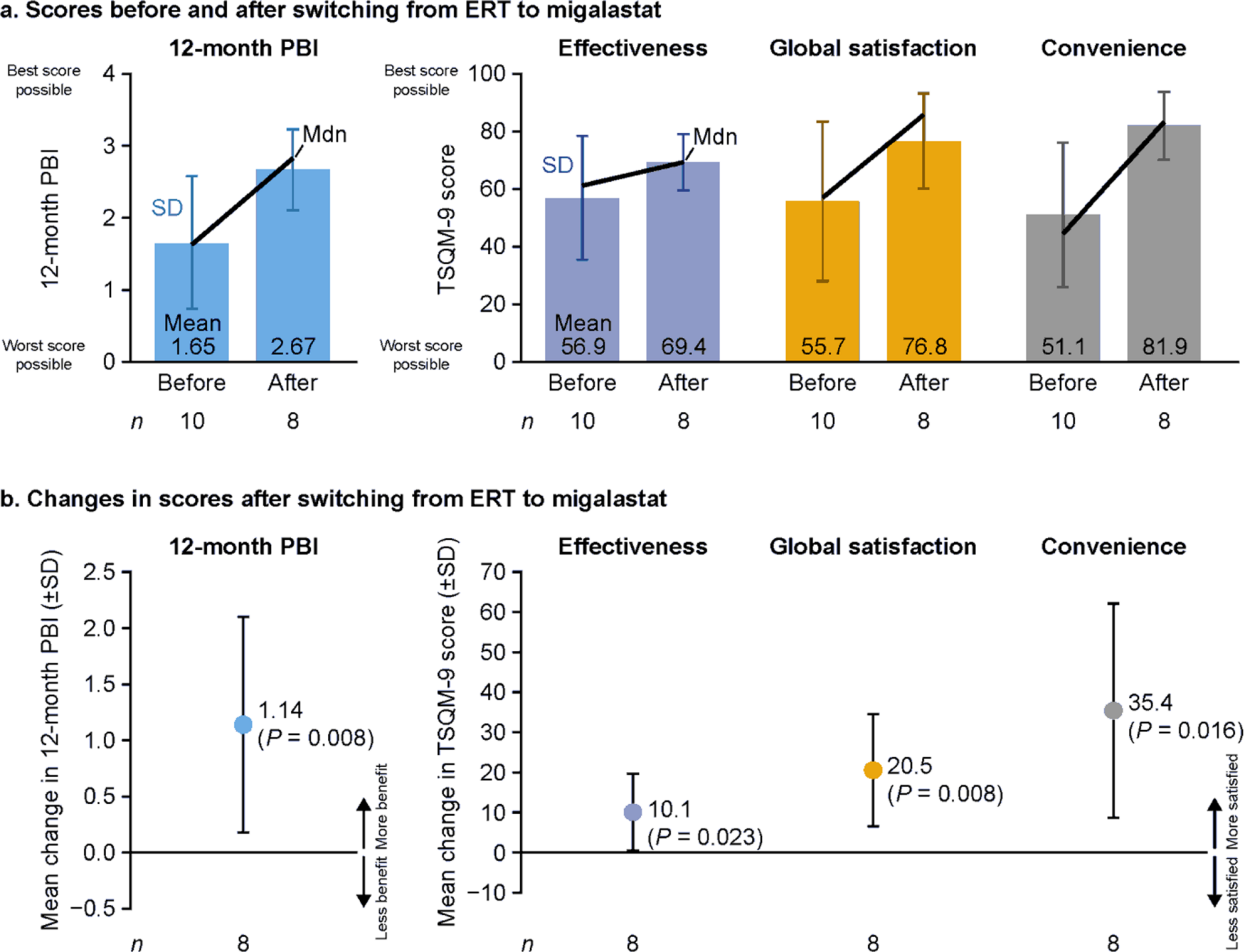
12-month PBI and TSQM-9 scores in patients switching from ERT to migalastat. Ten patients switched before the final study visit, of whom eight had data available before and after switching. Data for one additional patient who switched at the final visit (month 24) are not shown because post-switch values are not available. In panel **b**, p values are from a Wilcoxon signed-rank test. Mdn, median; PBI, Patient Benefit Index; SD, standard deviation; TSQM-9, Treatment Satisfaction Questionnaire for Medication-9

For patients who switched from ERT to migalastat at or after baseline but before month 24, mean (SD) 12-month PBI before switching from ERT to migalastat (1.65 [0.93], *n* = 10 with available data) was numerically lower than that for the overall population at 12 months (2.36 [0.91], *n* = 55) and 24 months (2.49 [0.94], *n* = 47). After switching to migalastat, 12-month PBI significantly increased by a mean (SD) of 1.14 (0.96; *P* = 0.008; *n* = 8 with available data; Fig. 5). In addition, all TSQM-9 domain scores significantly increased after switching from ERT to migalastat (*n* = 8), most notably convenience (Fig. 5).

### Additional endpoints and assessments

Analyses relating to the external validity of the PBQ and PBI are presented below. Results from all other exploratory endpoints are provided in the Supplementary Results.

Good completion rates for the PNQ-Fabry and PBQ suggested that the questions were well adapted and easy to answer. Significant positive correlations with TSQM-9 effectiveness and global satisfaction domain scores were observed for total PBQ (0.62 and 0.54, respectively) and 24-month PBI (0.63 and 0.61, respectively), where 1.00 indicates the strongest possible positive correlation (all *P* < 0.0001). Correlations of total PBQ and 24-month PBI with the TSQM-9 convenience domain score were lower (0.27 and 0.43, respectively) but still statistically significant (*P* < 0.0001). See Supplementary Table 5 for these analyses and for correlations between 6-month PBI and TSQM-9 domain scores.

Laboratory and echocardiography parameters were collected if assessed but were not study endpoints. Owing to the extent of missing information, particularly regarding the dates of assessments, no interpretation is provided (see Supplementary Results, including Supplementary Table 6).

## Discussion

SATIS-Fab was a prospective, non-interventional study conducted in France in which benefit, satisfaction and preferences associated with Fabry disease treatments were evaluated in patients with a migalastat-amenable *GLA* variant.

As anticipated, the SATIS-Fab study population was clinically heterogeneous. The population included patients with both classic and late-onset Fabry disease phenotypes, presenting at baseline with a range of Fabry disease signs and symptoms. MSSI scores ranged from 0 to 32, and while none of the patients had disease classified as severe based on the MSSI, 81.2% were already receiving treatment at baseline. This may have had an ameliorating effect on MSSI scores [35]. Moreover, although most of the population (60.9%) had the late-onset Fabry disease phenotype, disease progression in such patients is nonetheless still a serious concern [6]. In total, 82.6% of patients were prescribed migalastat at the baseline visit and all patients who changed their Fabry disease treatment during the study (after the baseline visit) switched from ERT to migalastat. Patient engagement with the SATIS-Fab study was good, as evidenced by the low number of discontinuations overall (ergo, the low number of discontinuations owing to the burden of study participation) and the good proportion of completed questionnaires; this is particularly notable given that the COVID-19 pandemic began during the study.

The main focus of the SATIS-Fab study was on patient-reported benefits, satisfaction and preference associated with Fabry disease treatment over the 2-year study (assessed using the PNQ-Fabry–PBQ–PBI methodology, the validated TSQM-9 and a single question, respectively). In this heterogeneous study population, most of whom were receiving migalastat, 92.3% of patients reported a treatment benefit at 24 months that was considered clinically relevant (24-month PBI score ≥ 1) and 30.8% a treatment benefit considered high (24-month PBI score ≥ 3) based on the needs and expectations they had expressed at baseline with the PNQ-Fabry. Additionally, treatment benefit over shorter time periods during the study (assessed with 6- and 12-month PBIs) remained relatively high and stable. These findings suggest that the Fabry disease treatments continued to meet patients’ needs and expectations throughout the 2-year period. In the subgroup of patients who switched from ERT to migalastat, treatment benefit (assessed with the 12-month PBI) and satisfaction (assessed with all three TSQM-9 domains) increased. Although only eight patients switched treatment, treatment benefit and satisfaction increases were significant. The mean change in PBI was, additionally, > 1; this may indicate meaningful treatment benefit. In the overall population, a preference for migalastat treatment was expressed by 72.5% (50/69) and 73.7% (42/57) of participants who responded at baseline and month 24, respectively. Collectively, continued or improved treatment benefit and satisfaction in the present study are consistent with high levels of real-world treatment adherence previously reported with migalastat [36].

There are limited Fabry disease-specific PRO tools. The PNQ-Fabry and associated PBQ and PBI are the only disease-specific tools to measure treatment benefit relative to patients’ perceived needs and expectations. To our knowledge, the SATIS-Fab study is the first to use the PNQ-Fabry and associated PBI to assess treatment benefit in patients with Fabry disease receiving ERT or migalastat in a real-world setting. Importantly, high levels of satisfaction apparent with the PBI, for the overall population and for patients switching from ERT to migalastat, were aligned with data from the TSQM-9, a clinically validated measure of treatment satisfaction. Additionally, the external validity of the PBI was confirmed by significant positive correlations between both the 24-month and 6-month PBI and the established, validated TSQM-9 effectiveness and global satisfaction domain scores (and the less marked, but still significant, correlation with the TSQM-9 convenience score). Notably, the magnitudes of the correlations may suggest that the PBI captures aspects of patient experience that the TSQM-9 does not. To summarize, the SATIS-Fab study findings indicate that the PBI is a relevant and useful tool for the evaluation of treatment benefit in patients with Fabry disease, as has been demonstrated in other chronic diseases [25–28]. The findings also emphasize the importance of patient–physician conversations about treatment for Fabry disease and tailoring management to patient needs and expectations to maximize their treatment benefit and satisfaction [16].

The assessments of treatment benefit, satisfaction and preference during the study were complemented with analyses of baseline data to explore whether distinct patient profiles exist for self-reported needs and expectations regarding Fabry disease treatment. The evidence for the existence of such profiles was mixed. On one hand, patients in the three clusters from the *post hoc* analyses based on clinical characteristics had slightly differing needs and expectations. Interestingly, there were no significant differences in 12- and 24-month PBI among these clusters, suggesting that treatment can provide benefit across different disease presentations. On the other hand, most patients in the prespecified analysis by PNQ-Fabry responses were in the same large cluster, which may indicate relatively similar treatment needs and expectations despite heterogeneous clinical characteristics. Alternatively, it is possible that the sample size was insufficient to distinguish small differences among groups using the components yielded by the PCA in the prespecified analysis. Planning sample size for clustering studies is certainly challenging because such analyses are exploratory and there is no universal approach to sample-size calculation. These difficulties become even more pronounced in rare-disease research.

The SATIS-Fab study had some limitations that should be considered when evaluating the study findings. Detection of true differences for the variables of interest may be hampered by the relatively short 2-year follow-up period and the heterogeneity of the study population. However, heterogeneity reflects clinical practice and provides insights that augment those from potentially more homogeneous clinical trials. Additionally, the study was observational and so did not modify physicians’ underlying clinical practice; thus, there may have been differences in the management of Fabry disease among patients. There was no randomization of treatment, resulting in an expected unbalanced distribution of patients by treatment group; however, the study was not designed to compare treatments directly. Only a small number of patients switched treatment during the study, limiting the conclusions that may be drawn from secondary analyses of treatment satisfaction and benefit before/after switching. While the data regarding clinical events and the laboratory and echocardiography data (see Supplementary Results) would have provided interesting context for the main findings about treatment benefit and satisfaction, they were not the main focus of SATIS-Fab. Investigators were not required to provide detailed laboratory or echocardiography data as these were not study endpoints, and, accordingly, there were many missing values. Moreover, the assessment dates were not recorded, only visit windows. Although these factors limit any conclusions that can be drawn, there are a number of published clinical trials and real-world studies that focus on laboratory or echocardiography outcomes [35].

## Conclusions

In conclusion, patients living with Fabry disease included in the SATIS-Fab study expressed a relatively high level of treatment benefit regarding their own needs and expectations, and this benefit remained stable over 2 years. Most patients received migalastat during the study, and treatment benefit was seen across a range of disease presentations in this clinically heterogeneous population. A switch in Fabry disease treatment from ERT to migalastat significantly increased patient benefit and satisfaction, although patient numbers for these analyses were small. The findings of the SATIS-Fab study indicate that the Fabry disease-specific PBI, which is derived from the PNQ-Fabry and PBQ, could be used to support shared decision making about Fabry disease treatment. The study findings also underscore the value of assessing parameters that are relevant to patients’ lives using a tool such as the French-validated PNQ-Fabry.

## Supplementary Information

Below is the link to the electronic supplementary material.

Supplementary Material 1

## Data Availability

Data sharing proposals and requests will be reviewed on a case-by-case basis. Requests for data should be addressed to Ali Hariri at ahariri@amicusrx.com. Requests will be reviewed by a medical steering committee.

## Abbreviations

α-gal A: α-galactosidase A
BMI: Body mass index
BPI: Brief Pain Inventory
CI: Confidence interval
CKD-EPI: Chronic Kidney Disease Epidemiology Collaboration
eGFR: Estimated glomerular filtration rate
ERT: Enzyme replacement therapy
Gb3: Globotriaosylceramide
LVH: Left ventricular hypertrophy
Lyso-Gb3: Globotriaosylsphingosine
MSSI: Mainz Severity Score Index
PBI: Patient Benefit Index
PBQ: Patient Benefit Questionnaire
PCA: Principal component analysis
PNQ-Fabry: Patient Needs Questionnaire-Fabry
PRO: Patient-reported outcome
SD: Standard deviation
SF-36v2^®^: Short Form-36 Health Survey version 2^®^
TIA: Transient ischaemic attack
TSQM-9: Treatment Satisfaction Questionnaire for Medication-9
