# Supplementary material for: Treatment-related benefit and satisfaction in patients with Fabry disease in France: insight into patients’ expectations and preferences from the prospective, non-interventional SATIS-Fab study
Source: Orphanet J Rare Dis. 2026 Apr 1;21:162. doi: 10.1186/s13023-026-04285-7 (PMC13097820; doi:10.1186/s13023-026-04285-7)
Supplement: Supplementary file 1 — Supplementary Material 1 [file 13023_2026_4285_MOESM1_ESM.docx]

Supplementary Materials

**Supplement to:** Lidove O, Masseau A, Pugnet G, Lacombe D, Dussol B, Bekri S, Hagège A, Martinez C, Fardini Y, Fouilhoux A and Noël E.
Treatment-related benefit and satisfaction in patients with Fabry disease in France: insight into patients’ expectations and preferences from the prospective, non-interventional SATIS-Fab study

Supplementary Methods

Assessments and data analyses

Patient Needs Questionnaire-Fabry

The questionnaire is provided in Supplementary Table 1.

Quality of life

Quality of life was assessed using the Short Form-36 Health Survey version 2^®^
(SF-36v2^®^), which measures generic health concepts relevant across age, disease, and treatment groups [1] and has been used in numerous studies of patients with Fabry disease [2]. The eight health domain subscales and the physical and mental component scores were transformed to T-scores based on US general population norms (mean 50, standard deviation 10) using the PRO CoRE scoring software (Optum). The standard form of the self-evaluated transition item was used (i.e. participants were asked about the change in their health, in general, over a 1-year period). Data from the SF-36v2^®^ were analysed descriptively.

**Supplementary Table 1** PNQ-Fabry questionnaire [3]

| How important is it to you that the treatment …  *Please tick a box for each statement* | Not at all important | Slightly important | Moderately important | Fairly important | Very important | Does not apply to me |
| --- | --- | --- | --- | --- | --- | --- |
| ensures you feel less tired | ☐ | ☐ | ☐ | ☐ | ☐ | ☐ |
| reduces the pain in your hands and feet | ☐ | ☐ | ☐ | ☐ | ☐ | ☐ |
| ensures you are less breathless when performing daily activities or with strenuous activities | ☐ | ☐ | ☐ | ☐ | ☐ | ☐ |
| reduces gastrointestinal disorders (nausea, pain, diarrhoea, constipation) | ☐ | ☐ | ☐ | ☐ | ☐ | ☐ |
| enables you to tolerate variations with heat and temperature better | ☐ | ☐ | ☐ | ☐ | ☐ | ☐ |
| reduces the intensity, frequency, or duration of painful attacks | ☐ | ☐ | ☐ | ☐ | ☐ | ☐ |
| enables you to continue working | ☐ | ☐ | ☐ | ☐ | ☐ | ☐ |
| enables you to cope with physical exertion better | ☐ | ☐ | ☐ | ☐ | ☐ | ☐ |
| enables you to live normally, as if you did not have Fabry disease (handicraft, housework, gardening, playing with your children, grandchildren) | ☐ | ☐ | ☐ | ☐ | ☐ | ☐ |
| enables you to maintain your social life (work, school, family, friends) | ☐ | ☐ | ☐ | ☐ | ☐ | ☐ |
| enables you to travel easily | ☐ | ☐ | ☐ | ☐ | ☐ | ☐ |
| enables you to have a better quality of life | ☐ | ☐ | ☐ | ☐ | ☐ | ☐ |
| ensures you are not dependent on other people on a daily basis | ☐ | ☐ | ☐ | ☐ | ☐ | ☐ |
| enables you to spend time with your family | ☐ | ☐ | ☐ | ☐ | ☐ | ☐ |
| enables you to stay fit for longer | ☐ | ☐ | ☐ | ☐ | ☐ | ☐ |
| prevents the onset of heart, kidney, or neurological problems | ☐ | ☐ | ☐ | ☐ | ☐ | ☐ |
| slows down the deterioration of your organs (kidneys, heart, brain) | ☐ | ☐ | ☐ | ☐ | ☐ | ☐ |
| enables you to feel good every day, even on days preceding or following treatment administration | ☐ | ☐ | ☐ | ☐ | ☐ | ☐ |
| does not cause side effects or adverse effects related to the medication | ☐ | ☐ | ☐ | ☐ | ☐ | ☐ |
| ensures you do not experience pain and tiredness returning on days before medication is administered | ☐ | ☐ | ☐ | ☐ | ☐ | ☐ |
| reduces the amount of medication that you are taking | ☐ | ☐ | ☐ | ☐ | ☐ | ☐ |

| How important is it to you to have a treatment …  *Please tick a box for each statement* | Not at all important | Slightly important | Moderately important | Fairly important | Very important | Does not apply to me |
| --- | --- | --- | --- | --- | --- | --- |
| that easily fits into your schedule and lifestyle | ☐ | ☐ | ☐ | ☐ | ☐ | ☐ |
| that you can take or administer on your own | ☐ | ☐ | ☐ | ☐ | ☐ | ☐ |
| that is easy to administer | ☐ | ☐ | ☐ | ☐ | ☐ | ☐ |
| that is administered orally (in tablet or capsule form) | ☐ | ☐ | ☐ | ☐ | ☐ | ☐ |
| with a short duration of administration | ☐ | ☐ | ☐ | ☐ | ☐ | ☐ |

Reproduced from Table 2 of Noël E, et al. Orphanet J Rare Dis. 2019;14(1):284 (<https://doi.org/10.1186/s13023-019-1254-7>), which is licensed under [CC BY 4.0](http://creativecommons.org/licenses/by/4.0/).

Note that the PNQ-Fabry is validated in the French language (see Supplementary Appendix of Noël *et al*. 2019) but has also been translated into English. PNQ-Fabry, Patient Needs Questionnaire-Fabry.

**Supplementary Fig. 1** Calculation of PBI by reference to PNQ and PBQ assessments at preceding visits

**
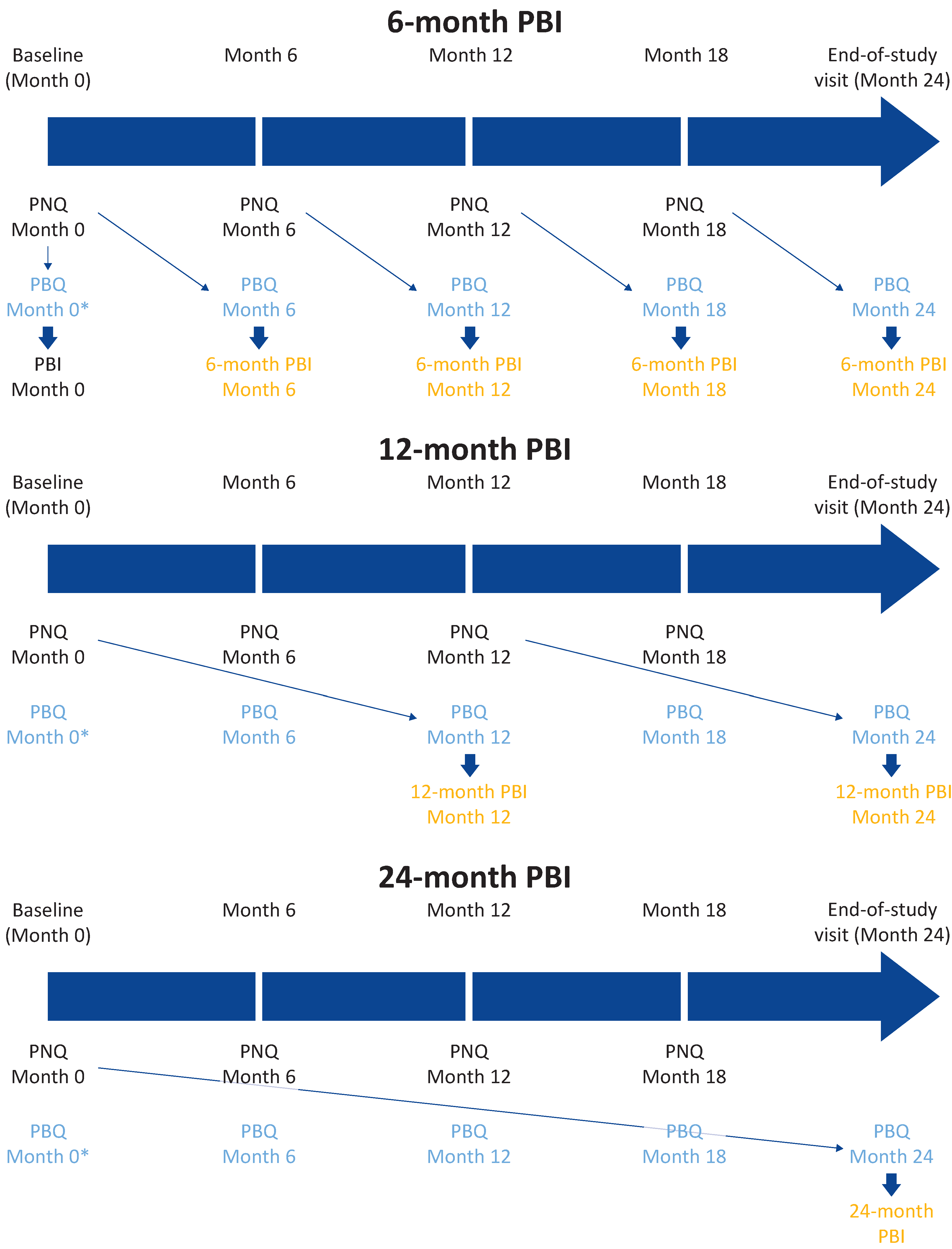
**

*Only recorded for patients who are already being treated with ERT or migalastat.

ERT, enzyme replacement therapy; PBI, Patient Benefit Index; PBQ, Patient Benefit Questionnaire; PNQ, Patient Needs Questionnaire.

**Supplementary Table 2** Protocol-specified clinical events

| Cardiac clinical events |
| --- |
| Myocardial infarction |
| New symptomatic arrhythmia requiring medication, direct current cardioversion, or interventional procedure (e.g., ablation, pacemaker, or defibrillator implantation) |
| Unstable angina defined by national practice guidelines accompanied by electrocardiographic changes resulting in hospitalization |
| Congestive heart failure requiring hospitalization or accompanied by elevated B-type natriuretic peptide |
| Cerebrovascular clinical events |
| Stroke (documented by a physician) |
| Transient ischaemic attack (documented by a physician) |
| Renal clinical and biological events |
| End-stage renal disease requiring dialysis or transplantation |
| Deterioration of the eGFR of more than 15 mL/min/1.73 m^2^ with GFR < 90 |
| Death due to any cause |

Wording is from the final study protocol.

eGFR, estimated glomerular filtration rate; GFR, glomerular filtration rate

Supplementary Results

Principal component analysis

**Supplementary Table 3** Principal component analysis of responses to the PNQ-Fabry

| Component | Main PNQ-Fabry questions associated with component | Label used to describe component* |
| --- | --- | --- |
| C1 | Q9, Q10, Q11, Q12, Q14 | Quality of social life |
| C2 | Q2, Q4, Q6 | Management of current symptoms |
| C3 | Q16, Q17, Q19 | Prevention of complications |
| C4 | Q1, Q3, Q8 | Resistance to physical exercise^†^ |
| C5 | Q23, Q24, Q25 | Easiness of treatment administration |
| C6 | Q15, Q18 | Fitness |
| C7 | Q22, Q26 | Disruption of daily life by the treatment |

The principal component analysis identified seven components (F1–F7) that explained 70% of variance in patients’ responses to the PNQ-Fabry. Because the methodology identifies which questions most strongly contribute to each component, some of the PNQ-Fabry’s 26 questions with negligible contributions are not represented here. *Based on the main PNQ-Fabry questions associated with the component. ^†^’Resistance’ refers to how the patient feels or how their body responds to physical exercise (based on Q1, Q3, and Q8).

C, component; PNQ-Fabry, Patient Needs Questionnaire-Fabry; Q, question.

*Post hoc* cluster analysis based on clinical characteristics

In a *post hoc* cluster analysis based on patients’ baseline clinical characteristics, three well-differentiated clusters were identified (Supplementary Table 4).

Cluster 1 (15 patients, 21.7%) had a small majority of patients with classic Fabry disease (53.3%), with a mean (standard deviation, SD) age of 47.1 (8.4) years and mean (SD) time since diagnosis of 11.9 (5.4) years. The mean Mainz Severity Score Index (MSSI) total score was the highest of any cluster (mean 19.6 [standard deviation (SD) 9.3]; range 1.0–32.0); neurological symptoms dominated – 73.3% had acroparaesthesia and the mean MSSI neurological subscore (7.7 [SD 5.7]) was severe – and 66.7% had left ventricular hypertrophy (LVH). Cluster 2 (27 patients, 39.1%) included the youngest patients, on average (mean [SD] age 41.7 [10.4] years), with a mean (SD) time since diagnosis of 5.3 (6.8) years, and most had late-onset Fabry disease (63.0%). The mean MSSI total score was the lowest of any cluster (11.3 [SD 6.5]; range 0.0–24.0), and although cardiac signs/symptoms occurred with a similar frequency to Cluster 1, no neurological pain was reported and 25.9% had acroparaesthesia. Cluster 3 (27 patients, 39.1%) consisted of older patients on average (mean [SD] age 64.3 [6.1] years) with the shortest mean time since diagnosis (3.9 [SD 5.2] years); most had late-onset Fabry disease (70.4%). The mean (SD) MSSI total score was 17.4 (6.1; range 6.0–32.0), and most experienced cardiac signs/symptoms (e.g. 81.5% had LVH and 40.7% had arrhythmia) but few had neurological pain (3.70%) or acroparaesthesia (11.1%).

**Supplementary Table 4** Overview of baseline characteristics and Fabry disease symptoms/complications by patient cluster

| Parameter | Cluster 1  *N* = 15 | Cluster 2  *N* = 27 | Cluster 3  *N* = 27 |
| --- | --- | --- | --- |
| Mean age, years (SD) | 47.1 (8.4) | 41.7 (10.4) | 64.3 (6.1) |
| Male, *n* (%) | 9 (60.0) | 19 (70.4) | 16 (59.3) |
| Mean BMI, kg/m^2^ (SD) | 25.1 (5.6) | 28.1 (6.8) | 26.5 (4.3) |
| Mean time since Fabry disease diagnosis, years (SD) | 11.9 (5.4) | 5.3 (6.8) | 3.9 (5.2) |
| Fabry disease phenotype,* *n* (%) |  |  |  |
| Classic | 8 (53.3) | 8 (29.6) | 7 (25.9) |
| Late-onset | 6 (40.0) | 17 (63.0) | 19 (70.4) |
| Other | 1 (6.7) | 2 (7.4) | 1 (3.7) |
| MSSI score,  mean (SD) / *median (range)*^†^ |  |  |  |
| General | 4.6 (3.3) / *4.0 (0.0–12.0)* | 2.0 (1.7) / *2.0 (0.0–8.0)* | 2.0 (2.4) / *1.0 (0.0–8.0)* |
| Neurologic | 7.7 (5.7) / *7.0 (0.0–16.0)* | 2.3 (2.7) / *1.0 (0.0–9.0)* | 2.4 (2.8) / *2.0 (0.0–10.0)* |
| Cardiovascular | 6.8 (6.1) / *8.0 (0.0–15.0)* | 5.4 (5.4) / *8.0 (0.0–15.0)* | 10.3 (5.5) / *11.0 (0.0–19.0)* |
| Renal | 0.5 (1.4) / *0.0 (0.0–4.0)* | 1.5 (2.5) / *0.0 (0.0–8.0)* | 2.7 (4.3) / *0.0 (0.0–18.0)* |
| Total | 19.6 (9.3) / *23.0 (1.0–32.0)* | 11.3 (6.5) / *12.0 (0.0–24.0)* | 17.4 (6.1) /*17.0 (6.0–32.0)* |
| BPI score,  mean (SD) / *median (range)*^‡^ |  |  |  |
| Pain severity | 4.2 (1.7) / *4.0 (1.0–8.3)* | 1.9 (1.7) / *1.8 (0–5.5)* | 2.3 (2.0) / *2.0 (0–6.5)* |
| Pain interference | 5.3 (2.1) / *5.0 (0–8.4)* | 1.5 (1.6) / *1.1 (0–6.3)* | 2.3 (2.0) / *1.3 (0–5.6)* |
| Fabry disease signs and symptoms / complications at baseline, *n* (%)^§^ |  |  |  |
| Cardiovascular |  |  |  |
| LVH | 10 (66.7) | 13 (48.1) | 22 (81.5) |
| Hypertension | 2 (13.3) | 6 (22.2) | 13 (48.1) |
| Arrhythmia | 2 (13.3) | 3 (11.1) | 11 (40.7) |
| Pacemaker | 1 (6.7) | 1 (3.7) | 7 (25.9) |
| Angina | 2 (13.3) | 2 (7.4) | 1 (3.7) |
| Heart failure | 0 | 1 (3.7) | 3 (11.1) |
| Valvulopathy | 2 (13.3) | 0 | 2 (7.4) |
| Myocardial infarction | 0 | 0 | 3 (11.1) |
| Implantable defibrillator | 1 (6.7) | 0 | 2 (7.4) |
| Neurological |  |  |  |
| Acroparaesthesia | 11 (73.3) | 7 (25.9) | 3 (11.1) |
| Stroke | 2 (13.3) | 3 (11.1) | 3 (11.1) |
| TIA | 4 (26.7) | 0 | 2 (7.4) |
| Neurological pain | 3 (20.0) | 0 | 1 (3.7) |
| Renal complications^¶^ | 1 (6.7) | 2 (7.4) | 3 (11.1) |
| Ophthalmological |  |  |  |
| Visual disturbance | 2 (13.3) | 2 (7.4) | 4 (14.8) |
| Verticillated cornea^‖^ | 0 | 2 (7.4) | 1 (3.7) |
| ENT |  |  |  |
| Hearing loss | 7 (46.7) | 4 (14.8) | 14 (51.9) |
| Tinnitus | 3 (20.0) | 5 (18.5) | 3 (11.1) |
| Vertigo | 6 (40.0) | 2 (7.4) | 1 (3.7) |
| Dizziness | 1 (6.7) | 1 (3.7) | 0 |
| Gastrointestinal |  |  |  |
| Abdominal pain | 5 (33.3) | 4 (14.8) | 0 |
| Diarrhoea | 4 (26.7) | 4 (14.8) | 1 (3.7) |
| Vomiting | 0 | 1 (3.7) | 0 |
| Early satiety | 0 | 1 (3.7) | 0 |
| Dermatological |  |  |  |
| Angiokeratoma | 6 (40.0) | 5 (18.5) | 5 (18.5) |
| Sweating disorders | 4 (26.7) | 4 (14.8) | 4 (14.8) |
| Psychosocial |  |  |  |
| Anxiety | 11 (73.3) | 3 (11.1) | 3 (11.1) |
| Depression | 8 (53.3) | 1 (3.7) | 2 (7.4) |
| Cognitive disorders | 2 (13.3) | 0 | 1 (3.7) |
| Pulmonary |  |  |  |
| Exertional dyspnoea | 2 (13.3) | 3 (11.1) | 3 (11.1) |
| Exercise intolerance | 3 (20.0) | 1 (3.7) | 3 (11.1) |
| Wheezing | 4 (26.7) | 0 | 0 |
| Cough | 2 (13.3) | 1 (3.7) | 0 |
| Skeletal |  |  |  |
| Osteopenia | 3 (20.0) | 0 | 1 (3.7) |

*Patients were determined to have a classic or late-onset phenotype based on their *GLA* variant status (using a Fabry disease phenotype/genotype database in which variants were categorized as noted in this table) and at the discretion of the study principal investigator. ^†^MSSI; composed of a total score (0–76 scale), with higher scores indicating more severe symptoms; based on four subscores: general (0–18 scale), neurologic (0–20 scale), cardiovascular (0–20 scale), and renal (0–18 scale) [4]. ^‡^BPI; consists of a pain severity score (0–10 scale) and pain interference score (0–10 scale), with 10 being most severe or highest level of interference [5, 6]. ^§^Investigators recorded Fabry disease signs and symptoms by selecting from protocol-defined terms or entering free text into a protocol-specified ‘other’ field, based on medical records and available laboratory data. ^‖^Coded by grouping all similar terms in the ‘other’ field.

BMI, body mass index; BPI, Brief Pain Inventory; ENT, ear, nose, and throat; LVH, left ventricular hypertrophy; MSSI, Mainz Severity Score Index; SD, standard deviation; TIA, transient ischaemic attack.

Based on their Patient Needs Questionnaire-Fabry (PNQ-Fabry) responses analysed according to the seven components from Supplementary Table 3, patients in the clusters had differing needs and expectations regarding their Fabry disease treatment. Patients in Cluster 1 placed greatest value on management of current symptoms, resistance to physical exercise, fitness, and quality of social life; those in Cluster 2 placed greatest value on (minimizing) disruption of daily life by treatment; and those in Cluster 3 placed greatest value on resistance to physical exercise, fitness, and (minimizing) disruption of daily life by treatment. Patients in Cluster 1 had the highest overall treatment needs and expectations compared with Cluster 2 and 3 (mean [SD] baseline PNQ-Fabry score: 90.1 [10.0] vs 75.4 [16.7] and 75.0 [19.0], respectively). Based on the proportions of patients responding ‘important’ or ‘very important’ to the three PNQ questions contributing to the factor labelled ‘prevention of complications’ (92.3–100% across the clusters and questions), nearly all patients wanted treatment to prevent or slow the complications of Fabry disease.

Treatment-related benefit and treatment satisfaction


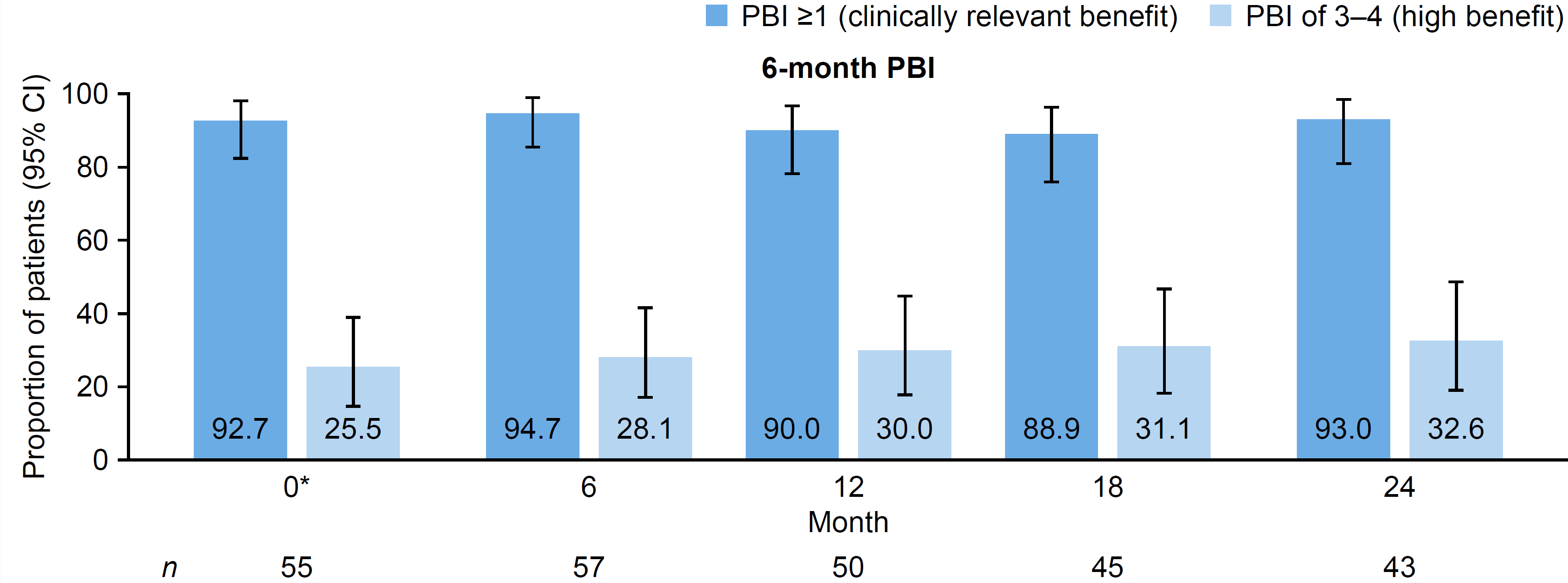
**Supplementary Fig. 2** Six-month PBI values during the study

Data are proportions of patients with clinically relevant or high benefit, with two-sided 95% CIs (Clopper–Pearson method).
*PBI for patients already treated before the baseline visit (calculated using PNQ-Fabry and PBQ responses at baseline; all other PBI values are calculated from PBQ responses at the time point shown and PNQ-Fabry responses from 6 months previously; see Supplementary Fig. 1).
CI, confidence interval; PBI, Patient Benefit Index.

**Supplementary Fig. 3** TSQM-9 score from baseline to month 24


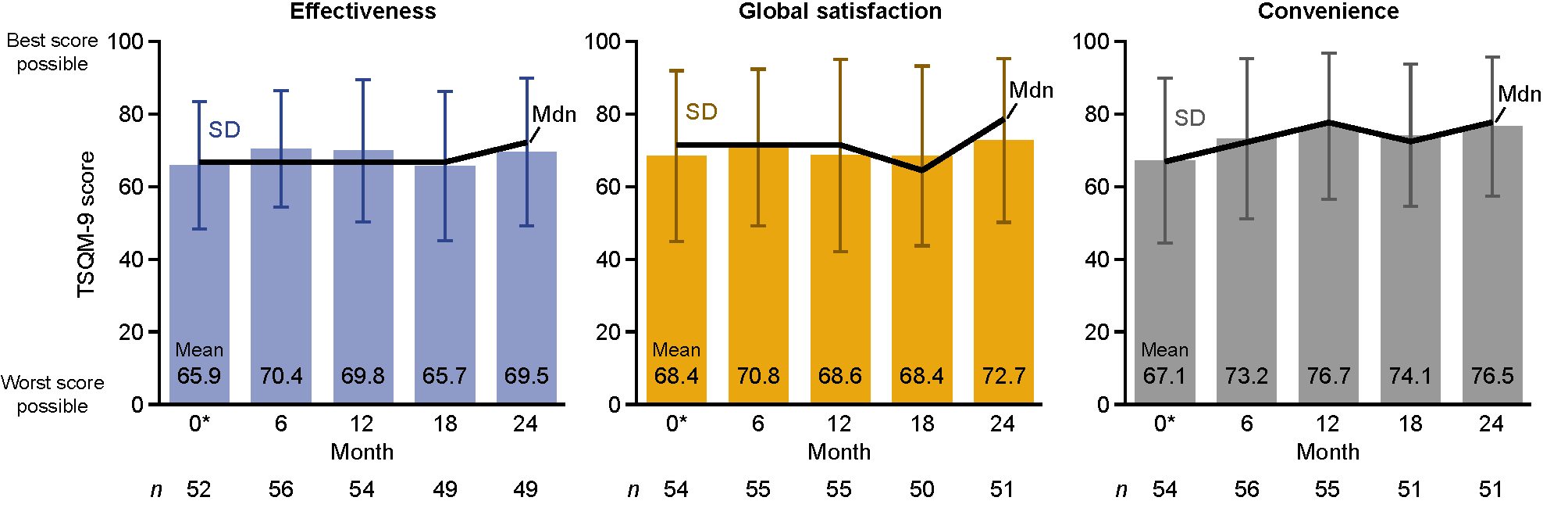


TSQM-9 comprises effectiveness, convenience, and global satisfaction domains, each with a possible score of 0–100; higher scores indicate greater satisfaction.

*Data from patients receiving ongoing treatment at baseline (*N* = 56).

Mdn, median; SD, standard deviation; TSQM-9, Treatment Satisfaction Questionnaire for
Medication-9.

Exploratory endpoints

External validity of the PBQ and PBI

**Supplementary Table 5** Pearson correlations between TSQM-9 and total PBQ, 6-month PBI, or 24-month PBI

|  | Total PBQ  (*N* = 345) | 6-month PBI (*N* = 252) | 24-month PBI (*N* = 109) |
| --- | --- | --- | --- |
| Mean (SD) | 43.1 (30.1) | 2.34 (0.91) | 2.31 (0.90) |
| TSQM-9 effectiveness score |  |  |  |
| *n* (for correlation) | 262 | 241 | 103 |
| Pearson correlation | 0.62 | 0.65 | 0.63 |
| p value | <0.0001 | <0.0001 | <0.0001 |
| TSQM-9 convenience score |  |  |  |
| *n* (for correlation) | 269 | 247 | 107 |
| Pearson correlation | 0.27 | 0.31 | 0.43 |
| p value | <0.0001 | <0.0001 | <0.0001 |
| TSQM-9 global satisfaction score |  |  |  |
| *n* (for correlation) | 267 | 245 | 107 |
| Pearson correlation | 0.54 | 0.60 | 0.61 |
| p value | <0.0001 | <0.0001 | <0.0001 |

*N*, number of data points available for Pearson correlation analyses; PBI, Patient Benefit Index; PBQ, Patient Benefit Questionnaire; SD, standard deviation; TSQM-9, Treatment Satisfaction Questionnaire for Medication-9.

Quality of life

At baseline, when assessed by SF-36v2^®^, patients living with Fabry disease had impaired quality of life in all subscales and component scores (i.e. mean scores <50) compared with a typical population of healthy individuals. Scores were relatively stable over time, e.g. the mean (SD) physical component and mental component scores were 43.4 (9.1) and 41.2 (10.7) at baseline (*n* = 64) and 43.7 (8.7) and 40.7 (11.6) at month 24 (*n* = 48), respectively. Based on the self-evaluated transition question, ‘Compared to 1 year ago, how would you rate your health in general now?’, the proportion of patients reporting stable general health (i.e. responding ‘About the same’) increased from 53.7% at baseline (*n* = 67) to 70.6% at month 24 (*n* = 51).

Fabry disease symptoms/complications

From month 6 to month 24, 28/69 patients (40.6%) had at least one new symptom or complication recorded. The most common newly reported individual symptoms and complications were neurological pain (8.7%), exertional dyspnoea (8.7%), anxiety (5.8%), and depression (5.8%).

Protocol-defined clinical events of interest

New protocol-defined clinical events of interest occurred in four patients (5.8%; aged 49–62 years, three male and one female) during the study period: three patients (4.3%) reported cardiac events (all new symptomatic arrhythmia), one patient (1.4%) reported a cerebrovascular event (stroke), and no patients reported renal events. No deaths occurred during the study.

Three patients who reported new clinical events of interest during the study had been receiving enzyme replacement therapy (ERT) at baseline; two switched to migalastat at the study baseline visit (at their own request or for lack of efficacy) and one continued to receive ERT. Of the patients who switched to migalastat, one had arrhythmia at the month 12 visit (and discontinued migalastat at the same visit because of pancreatitis) and one had arrhythmia at month 6 (and continued to receive migalastat for the remainder of the study). For the patient who remained on ERT, arrhythmia was reported at month 12 (and ERT was continued until the end of the study). The fourth patient was receiving migalastat at enrolment and was recorded as continuing migalastat at the baseline, month 12, month 18, and month 24 visits; stroke was reported at the final visit (month 24).

Pain

Overall, mean pain levels as measured by the Brief Pain Inventory (BPI) scale remained stable, although the variance was high. Mean (SD) values at baseline and months 6, 12, 18 and 24 were as follows. Pain severity: 2.55 (2.01), *n* = 65; 2.51 (2.30), *n* = 57; 2.63 (2.66), *n* = 55; 2.46 (2.64), *n*= 51; and 2.97 (2.56), *n* = 49. Pain interference: 2.68 (2.36), *n* = 62; 2.41 (2.51), *n* = 57; 2.63 (2.60), *n* = 54; 2.69 (2.36), *n* = 48; and 2.88 (2.54), *n* = 49. Mean (SD) changes at month 24 were 0.41 (2.06) for pain severity (*n* = 47) and 0.34 (2.02) for pain interference (*n* = 44).

Laboratory and echocardiography parameters

The SATIS-Fab study was not designed or powered to assess treatment effectiveness. Results of laboratory and echocardiogram assessments were recorded if performed.

Available laboratory data were assigned to a visit window, but assessment dates were not recorded, and data assigned to the baseline visit window could have occurred at any time before that visit. Levels of missing data were, overall, higher than those for the patient-reported outcomes. Supplementary Table 6 shows the estimated glomerular filtration rates; these were entered directly by the investigators (rather than being calculated centrally from serum creatinine levels). Given the limitations noted above, no interpretation has been made for these data.

Available echocardiogram data (specifically, end-diastolic interventricular wall thickness and end-diastolic posterior wall thickness) were included and assigned to a visit window only if they had taken place within the previous 6 months. Echocardiography parameters were poorly documented during the study and were missing for most patients at baseline (41/69 patients) and at month 24 (51/69 patients).

**Supplementary Table 6** eGFR data collected during the study*

|  | Baseline^†^  *N* = 69 | Month 6  *N* = 69 | Month 12  *N* = 69 | Month 18  *N* = 69 | Month 24  *N* = 69 |
| --- | --- | --- | --- | --- | --- |
| eGFR in mL/min/1.73 m²  Mean (SD) | (*n* = 57)  85.7 (26.6) | (*n* = 32)  79.3 (29.3) | (*n* = 37)  80.2 (24.4) | (*n* = 36)  84.3 (21.4) | (*n* = 46)  79.0 (28.0) |
| *n* (%)  15–29  30–59  60–89  ≥90 | 1^‡^ (1.8)  9 (15.8)  24 (42.1)  23 (40.4) | 1^‡^ (3.1)  7 (21.9)  12 (37.5)  12 (37.5) | 1^‡^ (2.7)  7 (18.9)  14 (37.8)  15 (40.5) | 0 (0.0)  4 (11.1)  17 (47.2)  15 (41.7) | 3^‡,§^ (6.5)  7 (15.2)  17 (37.0)  19 (41.3) |

No clinical events of deterioration in eGFR were reported.

*eGFR was not a study endpoint. eGFR values were entered directly into electronic case report forms by investigators rather than being calculated centrally from serum creatinine levels. Data were assigned only to a visit window, and no assessment dates were included. ^†^Data assigned to the baseline visit window could have occurred at any time before that visit. ^‡^Two patients who received ERT throughout the study had eGFR 15–29 mL/min/1.73 m², one at each recorded visit window (no measurement was recorded at month 18) and the other at month 24 only. ^§^One patient had eGFR 29 mL/min/1.73 m² at the month 24 visit window, having been treated with ERT before switching to migalastat at month 18. The treating physician wanted to conduct additional assessments to confirm the month 24 value. Had the value been confirmed, subsequent migalastat treatment would be considered off label; however, any subsequent eGFR assessments or treatment decisions would have been after the follow-up period had ended and, as such, were not recorded in the study database.

eGFR, estimated glomerular filtration rate; ERT, enzyme replacement therapy; SD, standard deviation.

# References

1. Ware JE, Jr. SF-36 health survey update. Spine (Phila Pa 1976). 2000;25(24):3130-9.

2. Arends M, Hollak CE, Biegstraaten M. Quality of life in patients with Fabry disease: a systematic review of the literature. Orphanet J Rare Dis. 2015;10:77.

3. Noël E, Dussol B, Lacombe D, Bedreddine N, Fouilhoux A, Ronco P, et al. Treatment needs and expectations for Fabry disease in France: development of a new patient needs questionnaire. Orphanet J Rare Dis. 2019;14(1):284.

4. Whybra C, Kampmann C, Krummenauer F, Ries M, Mengel E, Miebach E, et al. The Mainz Severity Score Index: a new instrument for quantifying the Anderson-Fabry disease phenotype, and the response of patients to enzyme replacement therapy. Clin Genet. 2004;65(4):299-307.

5. Jovanovic A, Miller-Hodges E, Castriota F, Takyar S, Howitt H, Ayodele O. A systematic literature review on the health-related quality of life and economic burden of Fabry disease. Orphanet J Rare Dis. 2024;19(1):181.

6. Tan G, Jensen MP, Thornby JI, Shanti BF. Validation of the Brief Pain Inventory for chronic nonmalignant pain. J Pain. 2004;5(2):133-7.
